# Intimate Relationships in Times of COVID-19: A Descriptive Study of Belgian Partners and their Perceived Well-Being

**DOI:** 10.5334/pb.1088

**Published:** 2022-01-10

**Authors:** Laura Sels, Sarah Galdiolo, Justine Gaugue, Marie Geonet, Pauline Verhelst, Claudia Chiarolanza, Ashley K. Randall, Lesley Verhofstadt

**Affiliations:** 1 Faculty of Psychology and Educational Sciences, UGent, BE; 2 Department of Clinical Psychology, UMONS, BE; 3 Psychological Sciences Research Institute, UCLouvain, BE; 4 Department of Dynamic and Clinical Psychology, and Health Studies – Sapienza University of Rome, BE; 5 Counseling and Counseling Psychology, Arizona State University, BE

**Keywords:** COVID-19, wellbeing, relationship quality, intimate relationships

## Abstract

How did couples in Belgium cope during the early phases of the COVID-19 pandemic? In this study, grounded in relationship science, we investigated in a descriptive manner several factors that could affect how couples perceived individual and relational wellbeing during this time. Specifically, we examined the associations between gender, sexual orientation, parental status, and relationship duration on participants’ self-reported individual and relational well-being after the first lockdown (more generally and more specific in response to COVID-19). Additionally, we investigated if relational well-being predicted perceived change in individual well-being from pre- to post-COVID-19 regulations. To test these hypotheses, self-report data was collected during the Summer of 2020 in both the Dutch and French speaking part of Belgium. Data from 679 participants suggested that individual and relational well-being only differed based on parental status (and not by gender nor sexual orientation). Importantly, parents reported lower relational well-being than participants without children, while participants without children reported higher perceived increases in depression. People that had been in a relationship for longer also reported lower relational well-being, but this relationship was explained by other confounding factors. Relational well-being buffered increases in individual distress that people perceived to have occurred pre-COVID-19 regulations to after COVID-19 regulations went into effect. These findings might inform practice and policy for individuals in a romantic relationship during the COVID-19 pandemic.

In response to the COVID-19 regulations, and the uncertainties that go along with it, many people are showing increases in emotional distress (Montemurro, 2020). Indeed, reviews of the available evidence show that people across the world report lower psychological well-being, and higher depression and anxiety than before the pandemic (Rajkumar, 2020; Vindegaard & Eriksen, 2020). COVID-19 patients report high levels of post-traumatic stress and depressive symptoms, and those in healthcare are also reporting higher levels of psychiatric symptoms than they did pre-pandemic (Vindegaard & Eriksen, 2020). Across the world, people report a variety of concerns and challenges, which can be summarized by the following: concerns for individual health and well-being, challenges to personal relationships, loss of future time perspective and adaptation to changes, and reactions of society, government, and media (Chiarolanza et al., under review). Not only are individuals coping with stress associated with contracting COVID-19, they are also coping with stress associated with governmental restrictions and lockdowns, which are negatively affecting their interpersonal relationships (e.g. Schokkenbroek et al., 2021b).

Strict government regulations in Belgium went into effect on the 18^th^ of March 2020 (Belgian Federal Government, 2020). Specifically, all restaurants and non-essential shops (e.g., clothes and furniture) were closed, people performing non-essential jobs were obliged to work from home or put on temporarily unemployment, social lives were restricted to people’s homes, and social distancing was required. A study based on 20,792 Belgian respondents showed that half of the respondents reported distress during the early days of this lockdown due to the consequences of the COVID-19 pandemic (Lorant et al., 2021). Since then, COVID-19 measures were relaxed during certain time spans and tightened during others, and uncertainty about how COVID-19 will evolve, continues.

Given our social worlds, in such times of continuous stress and challenge, people’s intimate relationships become increasingly important (Pietromonaco & Overall, 2020). When in distress, people tend to turn to and rely on those closest to them; and in adulthood, this is most often their romantic partner (Simpson & Rholes, 2012). Specific governmental restrictions taken in response to COVID-19, such as social distancing, can further increase the reliance on one’s intimate partner as a source of support. For instance, qualitative data collected across 20 countries found that participants reported feeling locked up at home, and feeling stressed due to: being always at home, a lack of time for the relationship, combining teleworking with coordinating school for their children, and lack of social leisure activities (Chiarolanza et al., under review). All this raises the question: how did people’s intimate relationships fare during the early phases of the COVID-19 pandemic?

The evidence available to date on this topic suggests that people’s intimate relationships did not invariantly suffer during these times, perhaps due to viewing one’s partner as a source of support (Randall et al., 2021). Effects on the relationship depend on several context, individual and relational factors (e.g., Overall et al., 2020). For instance, one study on American participants showed that overall relationship satisfaction did not change across the pandemic, however, this effect was moderated by relationship coping and relationship conflict (Williamson, 2020). In another study, some Chinese participants reported improved relationships with their partners, although very stressed individuals reported a decline in their relationship (Goodwin et al., 2020). Other studies did show an increase in intimate partner violence (Buttell & Ferreira, 2020; Moreira & da Costa, 2020), and relationship turbulence due to COVID-19 (Goodboy et al., 2021). In an American study, about one third of partnered individuals reported increased relationship conflict due to COVID-19 (Luetke et al., 2020).

In Belgium, one study found that participants reported more relationship stress in some areas (i.e. stress about conflict and diverging attitudes about the relationship), but not in others (i.e. stress about connectedness) during the first lockdown (Schokkenbroek et al., 2021b). Additionally, occurrences of domestic violence in Belgium during the first lockdown were observed in individuals who identified as lesbian, gay, or bisexual (De Schrijver et al., 2021), and stress about COVID-19 showed to positively predict verbal violence in partners (Schokkenbroek et al., 2021a). More recently, research conducted with Austrian partners found that relationship quality predicted better mental health and vice-versa (Pieh et al., 2020), which is also found outside of the COVID-19 pandemic. Indeed, relationship research has clearly shown that relationship functioning and individual mental health are strongly associated with one another (Whisman & Baucom, 2012).

## Present Study

The goal of this study was to understand how individuals in an intimate relationship living in Belgium perceived their well-being after the first lockdown. Specifically, Belgian individuals who were cohabiting with a relationship partner during COVID-19 were inquired about their individual and relational well-being, both general and specific in response to COVID-19. We aimed to investigate the potential roles of gender, sexual orientation, parental status, and relationship duration in determining participants’ individual and relational well-being after the first lockdown. Additionally, we investigated if relational well-being predicted perceived change in individual well-being from pre- to post-COVID-19 regulations. In this way, we aimed to obtain a comprehensive picture on the differential effects that COVID-19 might have on Belgian individuals in intimate relationships, depending on individual and context factors relevant to the relationship. Together, we aimed to replicate and extend existing research conducted in Belgium that showed negative effects of COVID-19 on specific aspects of individuals’ social and intimate relationships (Cauberghe et al., 2021; De Schrijver et al., 2021; Marchini, et al., 2020; Schokkenbroek et al., 2021a; Schokkenbroek et al., 2021b; Vermote, et al., 2021).

We focused on two indicators of individual well-being. Individual well-being was assessed more generally, by assessing participants’ global mental well-being. It was also assessed with an indicator focusing more explicitly on the perceived impact of COVID-19, by asking participants’ self-reported depression, anxiety, and stress, as they experienced it before the COVID-19 regulations (“before strict measures due to the coronavirus/COVID-19 on March 16, 2020 were introduced”) and after the COVID-19 regulations took place (“after strict measures due to the coronavirus/COVID-19 on March 16, 2020 were introduced”). Similarly, we focused on two indicators of relational well-being. Relational well-being was assessed more generally, by assessing participants’ global perceived quality of their relationship. It was also assessed with an indicator focusing more on the impact of COVID-19 and how people dealt as a couple with it, by asking participants how they and their partner coped together with COVID-19.

**Q1. Do reports of individual and relational wellbeing differ based on gender?**

Data collected across multiple countries has shown that men and women have been differentially affected by COVID-19. For example, in the UK, women have reported worse mental health outcomes than men (e.g., O’ Connor, et al., 2021; Proto & Quintana-Domeque, 2021). In one study, mental health declines in UK participants were twice as large in women compared to men (Etheridge & Spantig, 2020). In a Belgian study on psychological distress during the first lockdown, women also reported greater psychological distress than men (e.g., Lorant et al., 2021). Finally, a study across 26 countries revealed that women experienced significantly higher stress levels than men during quarantine (Kowal, et al., 2020).

Given data suggesting the differential impact of COVID-19 on men and women, many have speculated as to why this may be the case. First, women are more often frontline health workers (nursed, midwives, and community health workers) and health facility service staff than men (Thibaut & van Wijngaarden-Cremers, 2020). Second, women are disproportionally affected at home, having larger family and caring responsibilities (Waddell et al., 2021). Third, women are socially more affected, as they had larger social networks than men before COVID-19, and report increased loneliness due to COVID-19 (Etheridge & Spantig, 2020).

With regards to relational well-being, most international studies did not observe overall gender differences (e.g., Goodwin et al., 2020; Williamson, 2020), but women reporting greater household demands have reported increases in relationship problems and decreases in relationship satisfaction (Waddell et al., 2021). Related, women’s reports of intimate partner violence has also risen during COVID-19 (Moreira & da Costa, 2020).

Specific to Belgium, during the first lockdown, differences between men and women were found for specific indicators of relationship stress (Schokkenbroek et al., 2021b). Women experienced higher levels of relationship stress than men concerning diverging relationship attitudes with their partner (it is notable however, that women also reported higher levels of relationship stress in this domain before the lockdown). Additionally, women reported more stress during the first lockdown than before the lockdown because of relationship conflicts, while men did not show similar increases. Both partners did, however, report experiencing more stress due to feeling restricted in their relationship.

These studies give us reason to believe that partnered, Belgian individuals might differ in their individual and relational wellbeing after the first lockdown depending on gender.

**Q2. Do reports of individual and relational wellbeing differ based on sexual orientation status?**

Individuals who identify as members of the LGBQ (lesbian, gay, bisexual, queer) community have reported greater psychological distress and lower mental health and well-being following COVID-19 compared to heterosexuals (Buspavanich et al., 2021; Fish et al., 2021; Peterson et al., 2020). Besides experiencing more general distress, individuals who identify as a sexual minority experienced sexual-minority specific stressors, such as discrimination, which has detrimental effects on their mental health (e.g., Kneale & Bécares, 2020; Tung Suen et al., 2020). In Belgium, a recent study conducted with LGB+ participants showed that these participants experienced high levels of stress, alcohol and drug abuse, suicidal ideation and self-harming behaviour during the first lockdown (De Schrijver et al., 2021).

With regards to relational well-being, this study also showed that one third of Belgian LGB+ participants reported at least one incident of domestic violence. An international study on same-sex couples found that perceived COVID-19 threat negatively predicted relationship satisfaction (Li & Samp, 2021), but to our knowledge, no studies are available that explicitly compared relational well-being levels between heterosexual and sexual minority couples. Therefore, we wanted to investigate if individual and relational wellbeing in partnered, Belgian individuals differed based on sexual orientation status.

**Q3. Do reports of individual and relational well-being differ according to parental status?**

Couples experienced differential challenges due to COVID-19 and its regulations depending on parental status. While people without children might have experienced more boredom and isolation, parents on the contrary were overloaded (trying to combine multiplied household tasks with a lack of help) (e.g., Brown et al., 2020). For instance, research on family functioning based on a sample from Italy has shown that the pandemic has contributed to increases in parenting stress, with levels of household chaos (e.g. the organization of home spaces and routines and the quality of home atmosphere) predicting how much it increased (Spinelli et al., 2020). Additionally, a study across 26 countries revealed that individuals with children reported increased stress levels during the first lockdown compared with people living alone or with adults (Kowal, et al., 2020).

Partnered individuals without children might have had more time than ever to tend to, and support each other, but those with children have not necessarily been so lucky. Indeed, a study conducted in Spain found that childless couples reported increased levels of relational well-being (in terms of dyadic adjustment), while parents with children at home showed much more ambiguous patterns (Günther-Bel et al., 2020). As a consequence, we wanted to investigate if individual and relational wellbeing in partnered, Belgian individuals differed based on parental status after the first lockdown.

**Q4. Do reports of individual and relational well-being differ depending on relationship duration?**

Although both relationship status and age have shown to negatively predict stress and mental problems during the COVID-19 pandemic across different countries (e.g., Kowal, et al., 2020; Knepple et al., 2021), the potential role of relationship duration of partnered individuals on their well-being during COVID-19 has not been explored.

Historically, it has been thought that relationship quality and satisfaction show a curvilinear U-shaped trend, with a decline after the early years of the relationship, and an improvement later in life again (Anderson et al., 1983). However, recent research points at a general decline in relationship satisfaction over time (Proulx et al., 2017), which may be in part due to how couples cope with stressors across their life stages. For instance, couples in a later relational stage (e.g., couples whose children have already moved out) seem to have less disagreements, but also provide less support in their relationship when dealing with personal and relational stressors than young couples (Verhofstadt, 2009). Therefore, we wanted to investigate if relationship duration was associated with individual and relational well-being during COVID-19.

**Q5. Does relational well-being moderate the change in perceived individual well-being from pre- to post-COVID-19 regulations?**

There is a well-established link between the quality of our romantic relationship and our mental health (e.g., Whisman, 2013), and studies in Chinese and Austrian participants show that one’s relationship quality seems an important buffer for the negative effects of COVID-19 on people’s mental health as well (e.g., Goodwin et al., 2020; Pieh et al., 2020). In other international studies, specific interpersonal processes, such as dyadic coping (Randall et al., 2021) and perceived partner responsiveness (Balzarini et al., 2020) were observed to moderate the negative association between individual distress and relational well-being. In this study, we wanted to verify if in Belgian partnered individuals, relational well-being moderated the perceived change in individual well-being. Does the perceived effect of COVID-19 on people’s individual well-being depend on their relational well-being (in terms of the quality of their relationship and their perception of dyadic coping with COVID-19)?

To answer these questions, we relied on a one-time online survey focusing on people in an intimate relationship who cohabited with their partner during COVID-19. The data collection of this study occurred during the summer of 2020 (starting from the end of May and ending at the beginning of August), right after the first lockdown. While the strict measures of the first lockdown were relaxed at the end of May, homeworking was still recommended, and social distancing measures were still in place. Because of rising COVID-19 incidences, regulations became more strict again at the end of July.

## Method

### Participants and Procedure

This study was part of a larger pre-registered international study on the impact of COVID-19 on intimate relationships (see *https://osf.io/9hsdg* for more information). Given this study’s focus on the impact of COVID-19 on Belgian individuals, only these data are presented here. Institutional review board approval was obtained from Ghent University (ref: 2020/40) and University of Mons (no reference number available).

Potential participants were recruited across the Dutch and French regions of Belgium by survey distribution on various social media sites (e.g., Facebook), professional networking sites (e.g., LinkedIn), and personal networks. Data collection took place from May 19 (for the French speaking part) and May 25 (for the Dutch speaking part) to July 7 (for the Dutch speaking part) and August 7 (for the French speaking part) 2020. Country level COVID-19 restrictions went into effect on March 8, 2020. The first lockdown officially started the 18^th^ of March.

Participants were eligible to participate if they were 1) at least 18 years of age, 2) living in Belgium, 3) at least one year in an intimate relationship, and 3) living together with their partner.^1^ Interested participants were directed to the online survey link, which contained a copy of the informed consent and screening survey. A total of 2,160 participants accessed this online survey. If eligible, participants were automatically directed to the research survey. Participants were omitted based on 1) ineligibility for screening criteria, 2) having the French nationality, 3) responding “Yes” or “I don’t know” when asked if their partner also completed the survey, and 4) having more than 25% responses missing on the survey (based on their responses on quantitative questions). As a result, the final sample for this study included 679 participants.

Five hundred and two participants were considered Dutch Speaking (and were recruited in the Flemish part of Belgium, 74%), and 177 participants were considered French speaking (and were recruited in the Walloon part of Belgium, 26%). Across the entire 679 participants, 92% of the sample identified as women (*n* = 624), 7% identified as men (*n* = 47), 0.6% identified as non-binary (*n*= 4), 0.3% as gender fluid (*n* = 2), and two participants did not identify themselves. With regards to sexual orientation, 90.6% of the sample identified as heterosexual (*n* = 615), 4.4% identified as bisexual (*n* = 30), 3.5% as lesbian (*n* = 24), 0.7% as gay (*n* = 5), 0.3% as queer (*n* = 2), and 0.4% as other (*n* = 3). Three hundred twenty-two participants were married (47%), and 411 reported to have children (61%). On average, participants were 38 years old (SD = 12) and had been in a relationship for 14 years (SD = 12).

### Measures

All were validated questionnaires (except for the relationship duration questions) and validated Dutch and French translations were used when available.

#### Gender

Gender identity was assessed by asking participants to indicate with which category they identified themselves: men, women, non-binary, gender fluid, or other. Because of the low endorsements of the categories non-binary, gender fluid, and other (see participant section), these categories were excluded from analyses focusing on gender, yielding a sample of 671 participants.

#### Sexual orientation

Sexual orientation was assessed by asking participants to indicate their sexual orientation: heterosexual, bisexual, lesbian, gay, queer, or other. Given our interest in examining potential differences between those who identify as heterosexual and as sexual minority (Q2), we dichotomized response options to reflect *heterosexuals* and *sexual minorities*.

#### Relationship duration

Relationship duration was assessed by the following questions “How long have you and your partner known each other?” and “How long have you been in a relationship with your partner?” Participants indicated the number of years and months for each question. For ease, answers to these two questions were averaged to yield a total relationship duration score. Answers on these items correlated at *r* = .91.

### Individual well-being

#### General well-being

General well-being was assessed with the Warwick-Edinburgh Mental Well-being Scale (WEMWBS; Tennant, et al., 2007). The WEMWBS is a 14-item scale of mental well-being covering subjective well-being and psychological functioning. All items are positively worded (e.g., “I have been feeling optimistic about the future”) and rated on a scale from 1 = *none of the time* to 5 = *all of the time*. A sum score was created (ranging from 0 to a maximum of 70), with the Cronbach’s alpha in this study equaling .93.

#### Depression, Anxiety, and Stress pre- and post-COVID-19 regulations

Symptoms of depression, anxiety and stress, were assessed with the Depression, Anxiety, and Stress Scale-21 (DASS-21; Lovibond & Lovibond, 1995). Participants answered the DASS-21 twice. First, they were asked to respond to the 21 items reflecting on their pre-COVID-19 experiences before the lockdown regulations went into effect in March. Next, they were asked to respond to the 21 items reflecting on their post-COVID-19 experiences (after the regulations went into effect). The DASS-21 consists of 21 items assessing depression (7 items, e.g.: “I couldn’t seem to experience any positive feeling at all”), anxiety (7 items, e.g.: “I felt scared without any good reason”), and stress (7 items, e.g. “I found it difficult to relax”). Each was rated on a 4-point Likert scale ranging from 0 = *does not or never apply to me* to 3 = *applied to me very much, or most of the time*.

Sum scores were created for depression, anxiety, and stress levels, separately for pre- and post-COVID-19 scores (ranging from 0 to a maximum of 21). Cronbach’s alphas ranged from .85 to .93. In addition, difference scores were created, each time subtracting pre-COVID-19 scores from post-COVID-19 scores (and could thus range from –21 to 21). These explicitly assessed the perceived change in distress (depression, anxiety, or stress) from pre to post-COVID-19 regulations, with higher numbers representing increasing perceived distress. As shown in ***Table 1***, mean difference scores were positive, indicating an average increase in perceived distress scores.

**Table 1:** Descriptive statistics for key variables for men and women, heterosexual, and sexual minority participants, and conducted Mann-Whitney U tests.


	MEN		WOMEN						HETEROSEXUAL PARTICIPANTS			SEXUAL MINORITY PARTICIPANTS				

VARIABLE	*M*	*MDN*	*SD*	*M*	*MDN*	*SD*	*U*	P*	*M*	*MDN*	*SD*	*M*	*MDN*	*SD*	*U*	P*

General well-being	50.02	51.00	8.84	50.11	51.00	10.26	13900.50	.88	50.24	51.00	10.15	47.82	49.00	11.26	16168.00	.22

Depression pre-COVID	2.83	1.00	4.17	3.52	2.00	4.23			3.44	2.00	4.22	4.05	3.00	4.34		

Depression post-COVID	4.47	3.00	5.27	5.00	3.00	5.19			4.88	3.00	5.17	6.42	5.50	5.65		

Difference in depression	1.64	1.00	3.35	1.48	0.00	3.94	1409.20	.88	1.45	0.00	3.85	2.38	1.00	4.34	16348.00	.14

Anxiety pre-COVID	2.23	1.00	3.33	2.90	1.00	3.70			2.82	1.00	3.69	3.63	2.00	4.01		

Anxiety post-COVID	2.79	1.00	4.28	3.64	2.00	4.30			3.57	2.00	4.35	4.42	3.00	4.76		

Difference in anxiety	0.55	0.00	2.04	0.74	0.00	3.13	14480.50	.88	0.75	0.00	3.08	0.80	0.00	3.19	18890.00	.58

Stress pre-COVID	4.38	3.00	4.52	5.80	5.00	4.81			5.69	5.00	4.78	5.94	5.00	4.91		

Stress post-COVID	5.23	4.00	5.44	6.87	6.00	5.76			6.72	6.00	5.71	7.75	6.50	6.51		

Difference in stress	0.85	1.00	3.70	1.07	0.00	4.62	14075.00	.88	1.03	0.00	4.53	1.81	0.50	4.99	18167.50	.46

Perceived relationship quality	103.57	106.00	17.65	105.28	110.00	19.16	13112.50	.88	105.46	110.00	18.96	100.86	107.00	20.42	16687.50	.14

Dyadic coping	3.69	3.83	0.61	3.77	3.86	0.65	13524.50	.88	3.75	3.86	0.65	3.82	3.90	0.67	18452.50	.49


* p values were adjusted using the Benjamini-Hochberg’s false discovery rate adjustment.

### Relational well-being

#### Perceived relationship quality

Participants’ perceived relationship quality was assessed by the Perceived Relationship Quality Component Inventory (PRQC-Inventory; Fletcher et al., 2000). The PRQC Inventory measures six relationship components (love, passion, commitment, trust, satisfaction, and closeness), which together can be combined to yield a total perceived relationship quality score. The PRQC Inventory consists of 18 items (three items for each component) that are rated on 7-point Likert-type scales, ranging from 1 = *not at all* to 7 = *extremely*. Example items are “How happy are you with your relationship?” and “How committed are you to your relationship?”. A sum score was used (ranging from 18 to a maximum of 126), and Cronbach’s alpha equaled .96.

### Dyadic coping with COVID-19

Participants’ perceptions of how they and their partner deal with COVID-19 were assessed by the Dyadic Coping Inventory (Bodenmann, 2008; Ledermann, et al., 2010). The 37-item DCI assesses several forms of dyadic coping as perceived by the *self* (“What do I do when my partner is stressed?”), and in the *partner* (“What does my partner do when I am stressed?”). It measures four components of dyadic coping: supportive, delegated, negative, and joint (common) dyadic coping. Supportive dyadic coping consists of assisting the partner in his or her coping efforts through problem- and emotion-focused support (e.g., “my partner shows empathy and understanding”). Delegated coping consists of taking over responsibilities to reduce the partner’s stress (e.g., “My partner takes on things that I normally do in order to help me out”). Negative coping includes three aspects: hostile dyadic coping (support behaviors that are accompanied by disparagement, mocking, or sarcasm), ambivalent dyadic coping (reluctant, insufficient, or inefficient support), and superficial dyadic coping (insincere or undedicated support); items on negative dyadic coping are reversed scored for the total scale. Lastly, joint (common) dyadic coping consists of processes in which both partners participate more or less equally in order to handle stressful couple events (e.g. “We try to cope with the problem together and search for shared solutions”). Together, these four components load on and result in one total dyadic coping score.

Items were worded so that they were specific to COVID-19 (e.g., “What do I do when my partner is stressed about COVID-19”) and rated on a 5-point Likert-type scale, with 1 = *very rarely* to 5 = *very often*. Mean scores were calculated, ranging from 0 to 5. Cronbach’s alpha equaled .89.

Descriptive statistics for all key measures can be found in ***Tables 1*** and ***2***.

**Table 2:** Descriptive statistics for key variables for participants with and without children separately, and conducted Mann-Whitney U tests.


	PARTICIPANTS WITH CHILDREN		PARTICIPANTS WITHOUT CHILDREN					

VARIABLE	*M*	*MDN*	*SD*	*M*	*MDN*	*SD*	*U*	P*

General well-being	50.52	52.00	10.23	49.25	50.00	10.31	47710.50	.14

Depression pre-COVID	3.52	2.00	4.24	3.45	2.00	4.24		

Depression post-COVID	4.77	3.00	5.21	5.42	4.00	5.25		

Difference in depression	1.25	0.00	3.80	1.97	1.00	4.03	47921.50	**.01**

Anxiety pre-COVID	2.78	1.00	3.71	3.08	2.00	3.75		

Anxiety post-COVID	3.45	2.00	4.37	3.94	2.00	4.43		

Difference in anxiety	0.68	0.00	3.09	0.86	0.00	3.07	53345.00	.47

Stress pre-COVID	5.67	5.00	4.93	5.76	5.00	4.57		

Stress post-COVID	6.66	6.00	5.77	7.06	6.00	5.83		

Difference in stress	0.98	0.00	4.49	1.29	0.00	4.71	52652.00	.40

Perceived relationship quality	102.86	109.00	21.09	108.35	112.00	15.11	48048.50	**.01**

Dyadic coping	3.66	3.69	0.66	3.91	3.97	0.61	42601.50	**<.001**


* p values were adjusted using the Benjamini-Hochberg’s false discovery rate adjustment.

## Results

First, we investigated the potential impact of specific categorical characteristics (Q1: gender; Q2: sexual orientation, Q3: parental status) on participants’ individual and relational well-being (more generally and more specific in response to COVID-19). Individual well-being was assessed by participants’ general well-being score, and participants’ perceived changes in depression, anxiety, and stress scores going from pre- to post-COVID-19 regulations. Relational well-being was assessed by participants’ general relationship quality scores and their scores on their dyadic coping with COVID-19.

Before conducting the analyses, we tested homogeneity of variance and normality assumptions for the scores of the different subsamples on individual and relational well-being indicators. While equal variances could be assumed (according to Levene’s tests), normality assumptions were violated in the subsamples for almost all the variables. Because of the low sample sizes in some subsamples, we therefore decided to use non-parametric independent samples t-tests (Mann-Whitney U tests). For comparison, we also conducted parametric independent samples t-tests, which revealed no differences in findings.

Because of multiple testing, we decided to adjust p-values using Benjamini-Hochberg’s false discovery rate adjustment.

**Q1: Differences by gender**

When comparing men and women, we observed no significant differences in their individual or relational well-being (for exact statistics, see ***Table 1***).

**Q2: Differences by sexual orientation**

When comparing self-defined heterosexual and sexual minority participants, we found no significant differences in our variables of interest (after using Benjamini-Hochberg’s false discovery rate adjustment) (for exact statistics, see ***Table 1***).

**Q3: Differences by parental status**

When comparing participants with and without children, we observed no significant differences in their individual well-being in terms of general well-being and changes in perceived anxiety or stress (for exact statistics, see ***Table 2***). However, we did observe a significant difference for change in perceived depression, with participants without children reporting a higher increase in depression from pre-to post-COVID-19 regulations than participants with children. This corresponds to an effect size of .11, which is a small effect according to Cohen’s classification of effect sizes.

Follow-up analyses in which pre- and post-COVID-19-regulation scores of depression were compared, revealed that participants with and without children did not differ on depression pre-COVID-19 regulations (*Mdn_with children_* = 2 and *Mdn_without children_* = 2, *U* = 54462.00, adjusted *p* = .80), but that post-COVID-19 regulations, people without children tended towards more self-reported depression (*Mdn_with children_* = 3 and *Mdn_without children_* = 4), although this differences was not significant when taking into account the Benjamini-Hochberg’s false discovery rate adjustment (*U* = 50066.00, adjusted *p* = .09).

In terms of relational well-being, participants with and without children differed significantly from each other. Participants without children reported more perceived relationship quality and dyadic coping than participants with children. Both effects are considered small according to Cohen’s classification of effect sizes (.11 for perceived relationship quality, and .19 for dyadic coping).

**Q4: Relationship duration**

Next, we investigated if participants’ individual and relational well-being was associated with how long they had been in a relationship with their partner, using the same individual and relational well-being indicators as for the questions above. As normality tests rejected the normality assumption (both the Kolmogorov-Smirnov and Shapiro-Wilk tests showed *p* < .001), we decided to first apply Spearman rho correlation tests (for exact statistics, see ***Table 3***). In terms of individual well-being, participants that had been in a relationship longer, perceived lower increases in their stress and depression scores from pre to post-COVID-19 regulations. These effects are small in terms of effect sizes.

**Table 3:** Spearman rank-order correlation between relationship duration and individual and relational well-being variables.


VARIABLE	RELATIONSHIP DURATION	

	*R_s_*	P*

General well-being	.07	.08

Difference in depression	–.08	.**04**

Difference in anxiety	–.04	.36

Difference in stress	–.10	**.016**

Perceived relationship quality	–.14	**<.001**

Dyadic coping	–.18	**<.001**


* p values were adjusted using the Benjamini-Hochberg’s false discovery rate adjustment.

Perceived changes in anxiety were not associated with relationship duration. Follow-up analyses in which pre- and post-COVID-19 regulation scores, and their association with relationship duration were assessed separately, revealed that depression (*r_s,_* = –.11, adjusted *p* = .01) and stress scores (*r_s,_* = –.13, adjusted *p* < .01) post-COVID-19 regulations were negatively associated with relationship duration. Pre-COVID-19 regulations, only stress scores were negatively associated with relationship duration (*r_s,_* = –.09, adjusted *p* = .02).

However, in terms of relational well-being, a negative association with relationship duration was observed. This means that if couples had been in a relationship for longer, they perceived both their general relationship quality and their dyadic coping with COVID-19 to be lower. Again, these effects were small in terms of effect sizes.

As residualized change models are often advised for investigating change over time and can provide different results (e.g., Castro-Schilo & Grimm, 2018), we also applied multiple, hierarchical regression, in which post-COVID-19 regulation scores for individual well-being were the outcomes, and relationship duration was added as the predictor, while we controlled for pre-COVID-19 scores. These analyses allowed us to control for several potential confounding variables as well. Specifically, in a second step, general relationship quality, age of the participant, gender, sexual orientation, and having children were added as controls. Continuous predictors were centered around their means. These analyses (see ***Table 4*** for exact statistics) showed that participants reported lower depression after COVID-19 regulations went into effect when they had been in a relationship for longer. However, this association disappeared when controlling for general relationship quality, age of the participant, gender, sexual orientation, and parental status. The same was the case for stress, with longer relationship duration predicting less stress post-COVID-19 regulations, but again this association disappeared when relationship quality, gender, sexual orientation, age and the effect of parental status were controlled for. With regards to the control variables, relationship quality and age of the participant negatively predicted post-COVID-19 distress levels in terms of depression and stress. There was no significant effect of relationship duration on post-COVID-19 anxiety, and here, only relationship quality had a negative effect on anxiety levels.

**Table 4:** Residualized change models with multiple hierarchical regression, investigating the impact of relationship duration on depression, anxiety, and stress scores POST-COVID-19 regulations.


	OUTCOME VARIABLE																	

	DEPRESSION						ANXIETY						STRESS					

PREDICTOR OF INTEREST	MODEL 1			MODEL 2			MODEL 1			MODEL 2			MODEL 1			MODEL 2		

RELATIONSHIP DURATION	*B*	*SE*	*B*	*B*	*SE*	*B*	*β*	*SE*	*B*	*B*	*SE*	*β*	*B*	*SE*	*β*	*B*	*SE*	*β*

Intercept	4.98***	0.15		4.95***	0.59		3.58***	0.12		3.32***	0.47		6.76***	0.17		6.54***	0.68	

Pre-COVID	0.82***	0.04	.67	0.77***	0.04	.63	0.83***	0.03	.71	0.81***	0.03	.69	0.75***	0.04	.63	0.71***	0.04	.60

Relationship duration	–0.03**	0.01	–.08	0.00	0.02	.01	–0.01	0.01	–.04	0.00	0.02	.00	–0.05**	0.01	–.11	–0.02	0.02	–.05

Relationship quality				–0.04***	0.01	–.14				–0.03***	0.01	–.11				–0.04***	0.01	–.13

Gender				–0.02	0.59	.00				0.32	0.46	.02				0.56	0.68	.03

Sexual orientation				0.28	0.53	.02				–0.23	0.42	–.02				0.07	0.61	.00

Age				–0.05**	0.02	–.13				–0.02	0.02	–.07				–0.06**	0.02	–.14

Children				0.08	0.37	.01				–0.04	0.29	.00				–0.77	0.43	–.07

*R* ^2^	.46			.48			.51			.52			.42			.44		

*F*	277.20***			86.58***			341.89***			102.47***			234.10***			73.29***		


*Note*: * *p* < .05, ** *p* < .01, *** *p* < .001.

**Q5: Differences in effects of individual well-being, depending on relational well-being**

Finally, we investigated in a similar way if relational well-being moderated changes in perceived individual well-being from pre- to post-COVID-19 regulations. Specifically, we applied multiple, hierarchical regression, in which individual well-being scores post-COVID-19 regulations were the outcomes, and relationship well-being was added as the predictor (doing this separately for perceived relationship quality and dyadic coping). We controlled for scores pre-COVID-19 regulations, in this way modeling changes in distress levels. In a second step, we again controlled for age, gender, sexual orientation, relationship duration, and parental status, and centered continuous predictors.

These analyses showed that relational well-being in terms of perceived relationship quality negatively predicted depression, anxiety and stress post-COVID-19 regulations, also when controlling for age, gender, sexual orientation, relationship duration, and children (see ***Table 5*** for exact statistics). Thus, higher perceived relationship quality predicted higher individual well-being in terms of lower depression, anxiety and stress levels after post-COVID-19 regulations went into effect (controlling for pre-COVID-19 levels, and other potential confounds). All effect sizes were small, ranging from –0.16 to –0.18 (calculated by semi-partial correlations). For relational well-being in terms of dyadic coping with COVID-19, the effects were limited to depression. Specifically, higher levels of dyadic coping predicted lower depression after COVID-19 regulations went into effect, controlling for pre-COVID-19 depression scores, and when controlling for age, relationship duration, and having children. This effect size was low to medium, being –0.23 (calculated by semi-partial correlations). Higher levels of dyadic coping did not predict participants’ anxiety or stress levels post-COVID-19 regulations.

**Table 5:** Residualized change models with multiple hierarchical regression, investigating the impact of relational well-being (in terms of relationship quality and dyadic coping) on depression, anxiety, and stress scores POST-COVID-19 regulations.


	OUTCOME VARIABLE																	

	DEPRESSION						ANXIETY						STRESS					

PREDICTOR OF INTEREST	MODEL 1			MODEL 2			MODEL 1			MODEL 2			MODEL 1			MODEL 2		

RELATIONSHIP QUALITY	*B*	*SE*	*β*	*B*	*SE*	*β*	*B*	*SE*	*β*	*B*	*SE*	*β*	*B*	*SE*	*β*	*B*	*SE*	*β*

Intercept	4.99***	0.15		4.95***	0.59		3.59***	0.12		3.32***	0.47		6.76***	0.17		6.54***	0.68	

Pre-COVID	0.78***	0.04	.64	0.77***	0.04	.63	0.81***	0.03	.70	0.81***	0.03	.69	0.74***	0.04	.62	0.71***	0.04	.60

Relationship quality	–0.03***	0.01	–.12	–0.04***	0.01	–.14	–0.02***	0.01	–.10	–0.03***	0.01	–.11	–0.04***	0.01	–.11	–0.04***	0.01	–.13

Relationship duration				0.00	0.02	.01				0.00	0.02	.00				–0.02	0.02	–.05

Gender				–0.02	0.59	.00				0.32	0.46	.02				0.56	0.68	.03

Sexual orientation				0.28	0.53	.02				–0.23	0.42	–.02				0.07	0.61	.00

Age				–0.05**	0.02	–.13				–0.02	0.02	–.07				–0.06	0.02	–.14

Children				0.08	0.37	.01				–0.04	0.29	.00				–0.77	0.43	–.07

*R* ^2^	.47			.48			.52			.53			.42			.44		

*F*	286.06***			86.58***			354.38***			102.47***			235.68***			73.29***		

**Dyadic coping**																		

Intercept	4.98***	0.15		4.92***	0.60		3.58***	0.12		3.33***	0.47		6.75***	0.17		6.53***	0.69	

Pre-COVID	0.79***	0.04	.65	0.78***	0.04	.64	0.83***	0.03	.71	0.82***	0.03	.70	0.75***	0.04	.63	0.72***	0.04	.60

Dyadic coping	–0.70**	0.24	–.09	–0.88***	0.24	–.11	–0.29	0.18	–.04	–0.35	0.19	–.05	–0.47	0.27	–.05	–0.66*	0.27	–.07

Relationship duration				0.00	0.02	.00				0.00	0.02	–.00				–0.02	0.02	–.05

Gender				0.00	0.59	.00				0.32	0.47	.02				0.57	0.68	.03

Sexual orientation				0.53	0.53	.03				–0.06	0.42	–.00				0.33	0.62	.02

Age				–0.05**	0.02	–.12				–0.02	0.02	–.01				–0.06**	0.02	–.14

Children				0.05	0.37	.01				–0.10	0.30	–.01				–0.83	0.43	–.07

*R* ^2^	.46			.47			.51			.52			.41			.43		

*F*	277.97***			84.30***			342.58***			98.72***			226.38***			70.21***		


*Note*: * *p* < .05, ** *p* < .01, *** *p* < .00.

## Discussion

The COVID-19 pandemic is not only profoundly affecting people’s lives, but also people’s intimate relationships. In this study, we wanted to examine how Belgian individuals involved in an intimate relationship are faring during COVID-19. Specifically, we aimed to investigate if and how certain descriptive factors (mainly related to their intimate relationships) were associated with differences in the relational and individual well-being of Belgian partnered individuals during COVID-19. First, we investigated the potential impact of specific categorical characteristics (Q1: gender, Q2: sexual orientation, Q3: parental status) on participants’ individual and relational well-being (more generally and more specific in response to COVID-19). Next, we investigated if individual and relational well-being (general and specific to COVID-19) were associated with how long participants had been in a relationship (Q4: relationship duration); and finally, if relational well-being moderated perceived change in individual well-being from pre- to post-COVID-19 regulations (Q5: relational well-being).

No differences in individual and relational well-being were observed between men and women, or between heterosexual participants and sexual minorities. These findings are surprising, and somewhat inconsistent with past research (e.g., Fish et al., 2021), but might be explained by the low representation of men and sexual minorities in our sample. This underrepresentation might have limited our ability to observe effects. Further, it is notable that past research on the effects of COVID-19 on individual well-being also showed that differences between heterosexuals and sexual minorities reduced for people in a relationship (Buspavanich, et al., 2021).

We did observe differences between parents and non-parents in our sample. Specifically, we observed no significant differences in their individual well-being in terms of general well-being and changes in perceived anxiety or stress, but we did observe a significant difference for change in perceived depression, which seemed to be explained mainly by more self-reported depression post-COVID-19 regulations in participants without children. At the same time, in terms of relational well-being, people with and without children also differed significantly from each other: people without children reported more perceived relationship quality and dyadic coping than parents. These findings are in line with earlier findings (Günther-Bel et al., 2020), and suggest that the different circumstances and challenges people without and with children have to cope with, differentially impact their (individual and) relational well-being. Parents are often overloaded, having to balance work and childcare duties while coping with other disruptions in family life as well (Fontanesi, et al., 2020), which might put additional stress on the couple. In this regard, it has been shown that especially women are doing a disproportionally big part of their share, which contributes to household tensions and lower relationship quality (Biroli, et al., 2020; Waddell et al., 2021). Given that our sample consisted primarily of women, this might explain some of the findings.

At the same time, partners without children seem to experience more cohesion and connection (Günther-Bel et al., 2020), which might be due to enhanced proximity and shared activities, and an absence of other activities and social networks. It is interesting, however, that our participants without children reported a higher increase in depression than parents. A very tentative explanation at this point, is that this might have to do exactly with the loss of their social network and feelings of isolation (which again, affects women more than men; Etheridge & Spantig, 2020). Further, parents might have less time to ruminate about the pandemic and restrictions (which is associated with depression) than people without children, because parents have to focus on how to manage their children while schools are closed.

Further, we found some associations between relationship duration and individual well-being, with participants that had been in their relationship longer, perceiving lower increases in stress (and depression) from pre to post-COVID-19 regulations. However, these associations disappeared when controlling for relationship quality, age of the participants, and having children. This suggests that confounding factors accounted for this association, and that it did not had much to do with the relationship stage of the couple itself. For instance, age was negatively associated with post-COVID-19 distress levels (which is consistent with findings of other COVID-19 research; Horesh et al., 2020; Kimhi et al., 2020), which might be due to higher resilience at older age (Masten, 2002).

For relational well-being, the negative association observed between relationship duration and general relationship quality is in line with existing research (e.g. Proulx et al., 2017). However, also more specific dyadic coping with COVID-19 was negatively associated with relationship duration, which is expected according to some research (e.g. Verhofstadt, 2009) but not according to others (Berg & Upchurch, 2007). It seems that Belgian partners who were longer together employed less dyadic coping in dealing with this external stressor.

Finally, more general relational well-being in terms of perceived relationship quality, predicted less perceived increases in depression, anxiety, and stress after COVID-19 regulations went into effect. However, for relational well-being in terms of dyadic coping with COVID-19, the buffering effects were only observed for depression levels, and not for anxiety or stress. The finding that general relational well-being was associated with lower perceived increases in mental distress due to COVID-19 (in terms of depression, anxiety, and stress), is consistent with previous findings (Goodwin et al., 2020; Pieh et al., 2020). It is also consistent with broader relationship research showing strong associations between individual and relational well-being (Whisman, 2013; Whisman & Baucom, 2012). The finding that greater dyadic coping with COVID-19 was only significantly related to lower depression levels and not to anxiety or stress, is more surprising given existing research. In past research, dyadic coping was clearly associated with both general relational and individual well-being (e.g., Bodenmann et al., 2011), including depression (Bodenmann et al., 2004), anxiety (Regan et al., 2014), and stress (Meuwly et al., 2012). Further, this measure has been explicitly developed in the context of stress (Bodenmann, 1997). However, the dyadic coping inventory was developed to measure mainly how couples cope with stress in the context of their relationship, and although items were adapted to stress about COVID-19, this might have undermined its reliability. Further, we did not investigate subcomponents of dyadic coping, such as supportive, negative, or common coping, which might provide a different picture.

Indeed, this study is not without limitations. First, the cross-sectional design does not allow for causal conclusions, such as between individual and relational well-being. Relatedly, participants’ self-reported individual well-being in terms of the DASS before the COVID-19 regulations was assessed at the same time as participants’ self-reported individual well-being after the COVID-19 regulations went into effect; and actual information pertaining these participants before the COVID-19 regulations was absent. Indeed, the fact that people retrospectively report on their well-being for different time periods simultaneously, makes the data susceptible to recall and individual biases. However, measuring the same participants’ individual and relational well-being before COVID-19 started, was virtually impossible in the case of COVID-19. Thus, changes in scores on these measures should be interpreted as participants’ *perceived* changes in terms of anxiety, depression and stress, rather than actual changes in well-being. This is important in its own right, however, as it is people’s subjective feeling and experience of well-being that drives their behaviours (e.g., seeking professional counselling or therapy).

Second, participants self-selected into the study, and there was a large drop-out due to the length of the questionnaires. This might have resulted in a somewhat biased, homogenous sample. Because of the low amount of male participants and sexual minority participants, we warrant caution about a strong interpretation of the findings on gender and sexual orientation. Third, self-reports were used to assess mental health, as opposed to clinical interviews conducted by mental health professionals. Finally, the measurements took place in a specific period of COVID-19, when restrictions started to loosen progressively, and findings might have been different for other periods during the COVID-19 pandemic. However, we do think that measuring experiences throughout different periods during the COVID-19 pandemic, such as during lockdowns (e.g. Schokkenbroek et al., 2021b) and after, is needed to provide a more comprehensive picture of the impact of COVID-19 on people’s intimate relationships.

Finally, this study relied on participants’ individual reports of their relationship well-being, and not on reports from both partners within a couple. The choice for an individual rather than dyadic data-collection approach was mainly based on ethical, practical, and statistical reasons (e.g., the possibility of anonymization, and no dependencies between observations due to partners being part of the same couple).

Despite these limitations, this study provides useful information on how Belgian couples are coping with COVID-19, which might inform practice and policy during the still lasting pandemic and its associated confinements. Specifically, the results demonstrated the differential effects for couples with and without children and suggests that the quality of intimate relationships in families might be suffering due to the COVID-19 regulations and its consequences. Additionally, lower general relationship well-being and coping was observed for couples that were together longer. At the same time, the quality of intimate relationships plays a crucial role in people’s mental health and well-being; and in our study, relational well-being was indeed associated with less perceived increases in depression, anxiety, and stress during COVID-19. The important buffering, or on the contrary harmful role, that intimate relationships can play in people’s well-being during these times should therefore not be overlooked.

Concretely, mental health workers and policy makers may put more effort -either preventative or curative- into fostering high-quality intimate relationships, for example by highlighting the mutually supporting role of partners, especially in times where the partner is often the only source of support. To this end, not only a focus on strengthening the individuals’ relationships by calling on the individuals themselves, but also by addressing constraints of the ecological niche couples are embedded in, might be necessary to improve partners relational and individual outcomes. For instance, special attention could be paid to specific subgroups, and interventions tailored to these subgroups. For parents, interventions that lighten their responsibilities might be helpful, in order to allow them to invest more time and energy in their intimate relationship At the same time, couples without children seem to have less relational, but more individual needs in times of stressful events like COVID-19, and it might be worthy to explore how both relationship partners can help each other dealing with depressive feelings resulting from restricted life circumstances.

## Data Accessibility Statements

The data and corresponding code to carry out the analyses in the manuscript can be found on https://osf.io/u4ymg/. This research was supported by funding from the American Psychological Association’s Office of International Affairs (PI: Randall).

## Footnotes

Inclusion criterion were determined by the international project this study. For more information see Randall et al. (2021).
